# Examining the Relationship between Paternal Mental Health and Informal Support Networks: Reflections on the Impact of the COVID-19 Pandemic

**DOI:** 10.3390/ijerph191912751

**Published:** 2022-10-05

**Authors:** Ernestine Gheyoh Ndzi, Amy Holmes

**Affiliations:** York Business School, York St John University, York YO31 7EX, UK

**Keywords:** fathers’ mental health, COVID-19, fatherhood, pandemic, wellbeing, informal support

## Abstract

Paternal mental health remains an under-researched area in the UK. Consequently, father-focused formal and informal support provisions fail to address the complex emotional and psychological wellbeing needs of fathers. Drawing on data from twenty semi-structured interviews with fathers in the York area, this study seeks to better understand how access to and participation in informal support networks is influenced by gendered perceptions and the impact hegemonic perceptions of masculinity have on fathers’ access to support prior and during the COVID-19 pandemic. The findings demonstrate that fathers internalise stereotypical masculine tropes, such as stoicism, which prevent them from actively seeking support. While fathers value informal support network, they generally struggle to engage in mental health talks. The COVID-19 lockdown exacerbated fathers’ struggles to access informal support or prioritise their mental health. Fathers felt the pandemic presented a unique challenge that only people that became parents at the time understood. This meant that fathers could not rely on their parents or other parents who did not have similar experiences of the COVID-19 pandemic. This paper aims at challenging structural and cultural barriers that inhibit fathers’ participation in informal support networks, and to promote more meaningful, supportive engagement with peer groups.

## 1. Introduction

Mental health is a prominent public health issue and policy concern in contemporary society, with approximately one in every six people in the United Kingdom (UK) receiving a diagnosis during their lifetime [1]. According to this study, a fifth of men (19.5%) will receive a diagnosis during adulthood [1]. As such, it is essential to understand the factors that influence male mental health and wellbeing, particularly with fatherhood and the expectations this role places upon men. This paper explores the impact of the COVID-19 pandemic on fathers’ access to mental health support, particularly from informal support networks such as those made up of the father’s partner, family, and friends or peers. Using semi-structured interviews with fathers in the York area, the study illustrates the role of the gendered parental division of labour in creating barriers that fathers must navigate to form meaningful relationships with other parents. Although many families adopt a more egalitarian approach to parenting, traditional gender norms and embedded hegemonic masculine values [2] continue to influence the perception of many fathers who take an active role in parenting their children and, as a result, undermine their attempts to establish peer support networks. Coupled with the COVID-19 pandemic and subsequent lockdown regulations, socio-cultural tropes around stoicism and masculinity further isolate fathers who are experiencing poor mental health and discourage them from seeking support [3]. The paper further demonstrates that barriers to accessing paternal mental health support are not merely the product of the pandemic, but of cultural, occupational, and structural factors that reinforce the traditional, gendered, parental division of labour.

In the United Kingdom, traditional gender norms emphasise the father’s role as breadwinner within the heterosexual nuclear family [4]. Although these attitudes towards perceived gender differences in parenting persist [5], the contemporary fatherhood ideal encourages many fathers to be increasingly more actively involved in the lives of their children [6]. Alongside being more invested in their child’s life, contributing significantly to housework and equitable co-parenting were characterised as qualities of the ‘new fatherhood ideal’ [7]. This redistributed parental division of labour tends to address the dual burden experienced by mothers who work and act as primary carers for their children and, ultimately, be more productive for women as they return to the workforce [8]. However, the transition to fatherhood can be challenging and cause psychological distress [9], highlighting the importance of support for fathers. Despite rising concerns about paternal mental health [10], perinatal mental health services in the UK are still primarily focused on mothers, with limited support for fathers [11]. Research suggests that this lack of formalised support for fathers could also link to isolation from informal support groups and socio-cultural norms related to masculinity and the parental division of labour [10,11].

Concerning fatherhood, discourses of masculinity refer to the linguistic, structural composition, and expression of internal knowledge, ideological views, and social cognitions in the context of the gendered division of labour [12]. Drawing upon the work of Connell and Messerschmidt [2], it is evident that hegemonic discourses of masculinity produce and reinforce assumptions about men’s role as parents. Analogous to this, there is an urgent need to address how the discursive construction of gender and hegemonic masculine norms dissuade fathers from seeking mental health support [13]. For example, endorsements of the gendered dichotomy of emotionality, whereby women are permitted to freely express their emotions and men are urged to adopt stoicism and emotional restraint [14], are pervasive in male research samples across ages [15], culture [16], and occupational demographics.

In Western society, unyielding self-reliance and the stoic endurance of suffering are regarded as defining traits of masculinity [14], with emotionalism rejected as a feminine trait that is incongruent with masculine ideals. This inherently solidifies the inextricable link between men’s mental suffering and their fundamental reluctance to speak out about their distress. Further, restricted emotionality, which refers to deliberately suppressing or inhibiting emotional displays [17], has been consistently linked to negative attitudes towards help-seeking [18], meaning that men are more reluctant to engage with support services. Vogel et al. [19] provided empirical evidence that rigid conformity to conventional gender norms, such as the suggestions that ‘*real* men don’t cry’ and to ‘man up!’, intrinsically promoted self-stigmatisation amongst men who internalised these values. Previous research has linked self-stigma with decreased self-esteem, worth, and efficacy [20], with further research associating these factors with low treatment adherence and negative help-seeking behaviours [21].

Furthermore, fathers are often under-supported because they are systematically depicted as “part-time” caregivers who provide additional support and relief to mothers [22,23]. These perceptions are thought to be internalised by many fathers, who encounter gendered beliefs in healthcare and occupational settings during pregnancy and childbirth [24]. Within these interactions, men are often conceptualised as expendable and secondary in the child-bearing endeavour, disparaging their equal importance as a parent [25]. Collectively, this research is evidence of the role of societal discourse in promoting adherence to gendered parental roles and masculine narratives that dissuade men from seeking help. However, it is worthwhile to consider that since discourse corroborates the stigma and crisis attached to male mental health, this may also offer a productive pathway to dismantling it.

Prior to the COVID-19 pandemic, research on fathers illustrated the overwhelming nature of parental anxieties, specifically concerning the health of the partner and child [26]. Fathers perceived their relationship with their partners as their most important relationship within the informal support network [10]. This partnership was put under immense pressure with the introduction of a new child, influencing how fathers utilised informal and formal support networks when the dynamic of the relationship with their partner changed [26]. Many fathers who returned to work following paternity leave had less time to spend with their children, which some partners begrudged, creating tension within the partnership [27]. Furthermore, the lack of resources and support from health services meant that most fathers felt underprepared to involve themselves, which sometimes caused further conflict with their partners. Therefore, the tension on the fathers’ behalf was due to feelings of exclusion and dismissal [28], frequently creating additional strains within the relationship. As a result, fathers might alleviate the strain by turning to other members of the informal support network, such as friends and family. 

Research suggests that many fathers do not confide in their partners about their mental health challenges [26] because they perceive their partner’s experiences to be more challenging than their own and therefore do not want to trouble them further. Furthermore, many fathers frequently minimise their own difficulties in relation to the issues their partners experienced [10], supporting the idea that fathers perceive their partner’s physical and mental health as more important than theirs. For this reason, the fathers tend to deal with their struggles alone or by seeking support from people outside their relationship.

Support from other informal networks, such as family and friends, can increase positive mental health outcomes for the father, such as a reduction in anxiety [29]. However, fathers have often noted that the support they need is not always available, and they often receive irrelevant advice [26]. Informal support from family and friends frequently resulted in casual conversations, which may not be helpful [26]. Friends who are not parents relate less to conversations on parenthood, leaving fathers with non-parent friends struggling to find support from this network. Instead, valuable conversations about parenthood often occurred during collaborative activities, such as sports [10], where the casual environment encouraged light-hearted discussions amongst the participants. Casual conversations and collaborative activities were noted to provide a distraction from mental health struggles. A man’s reluctance to address mental health problems is often guided by the fear of negative assumptions and perceptions by those in the informal support network [26]. This suggests that fathers internalise hegemonic masculine ideals about themselves, which they feel must be maintained privately and publicly, as they believe that others also hold these ideals. Instead of engaging in deep talk about mental health, then, fathers keep these challenging topics to themselves.

The isolation during the pandemic was reported not to negatively affect the fathers’ relationship with their partners [30]. Instead, it was found that fathers gained a more profound understanding of their partner, aiding their provision of support. Furthermore, their mental health was said to have either improved or remained the same throughout the lockdowns [30]. 

Research indicates that, outside of the relationship with their partner, the informal support network of fathers acts as an additional buffer to negative mental health [31], including symptoms of depression and anxiety. During the pandemic, social contact was greatly restricted by law, resulting in the loss of physical contact with the informal support network outside of their partners for some fathers. This created feelings of isolation for some new parents, with the socialisation restrictions perceived as the most negative effect of the pandemic [32]. Many fathers tended to restrain visiting contacts, even when abiding by new restrictions, to avoid risking their partner’s and child’s health [33]. This meant that many fathers lost their in-person informal support network and could not take time away from the stresses of parenthood to engage in collaborative social activities. The inability to alleviate or distract oneself from stressors negatively impacts mental health, which was arguably the case for many fathers during the pandemic. While research conducted during the pandemic shows that some mothers’ mental health declined when they did not receive support from their informal network [34], no comparative research has been conducted on fathers. This demonstrates the value of researching fathers’ experiences and understanding the impact of the pandemic on paternal mental health and wellbeing.

During the COVID-19 lockdown, it was assumed that mothers worried more than fathers about the breakdown of the informal support network [35]. However, this is not to say that the breakdown of their informal networks does not impact fathers, as this is an under-researched area, and recent scholarship has not acknowledged the numerous reasons why fathers may not report concerns about their friendships or peer groups. Additionally, while the findings from van den Heuval et al. [35] were insightful, the study was based in The Netherlands, and it is essential to understand fathers’ experiences in the United Kingdom in the context of the different support systems and cultures. 

The study aims to explore how, in the York area, the breakdown in informal support networks may have contributed to some fathers’ existing mental health concerns during the COVID-19 pandemic. The study also explores the role of hegemonic, masculine cultural norms in shaping these fathers’ experiences of and access to informal support networks. Darwin et al. [10] suggested that many fathers use informal and casual support from other parents and seek parenting reassurance from other family members, thus relying upon their social networks to maintain their wellbeing. Consequently, it was essential to understand the level of dependency of fathers in the York area on informal support networks and the impact of the COVID-19 lockdown in the UK, where in-person social contact was banned. 

## 2. Materials and Methods

The study adopted a qualitative research method. Data were collected from twenty semi-structured interviews with fathers in the York area, exploring the impact of the COVID-19 pandemic on paternal mental health. Most participants (12) were recruited through social media platforms, such as Twitter, Facebook, and Instagram. Eight participants were recruited using a snowball and convenience sampling method [36]. This sample was selected for its geographical convenience. Interviews were conducted online via Zoom and lasted an average of fifty minutes. All the research participants were employed in the UK, with nine participants working in academia or research and eleven employed in occupations such as the army, the police, healthcare, education, and administration. Fifteen respondents identified themselves primarily as ‘White British,’ two as ‘Black British,’ and three participants did not disclose their ethnic backgrounds. Regarding experiences of fatherhood, fourteen were fathers prior to the pandemic, with five having their second child during the lockdown period. Additionally, five became fathers for the first time during the pandemic. 

The interview schedule consisted of six open-ended questions intended to cover three key areas: (1) father’s experience of transitioning into parenthood, (2) experiences of fathers accessing informal support, and (3) the impact of the COVID-19 pandemic on their ability to access relevant informal support and the role that norms regarding masculinity play in access to informal support systems. The interview sought to understand how informal support networks impacted the father’s mental health during the COVID-19 pandemic. To better understand the relationship between paternal mental health and the COVID-19 pandemic, a thematic analysis of the transcripts was conducted using the NVivo software to establish and code emergent themes. This was important to ascertain the impact of the pandemic on paternal mental health, with access and level of informal support networks emerging as the most pertinent avenue for exploration in the context of this paper. Identifying the role of informal support networks allowed for a better understanding of how men interact with their peers and how these friendships promote paternal wellbeing.

Although the study makes a valuable contribution to the field of paternal mental health studies, there are some key considerations to be made of the project: data were collected between April and June 2021, offering a vignette of fathers’ experiences during the pandemic. Participants were also employed mainly in York, meaning the findings are not generalizable across socio-economic groups and regions. These limitations are discussed in turn, before reflecting on the value of the contributions that this small, geographically specific study can make to the study of fatherhood.

The study offered valuable insights into the impact of the pandemic on paternal mental health. However, data collection were conducted between April and June 2021, and there have not since been opportunities to re-interview the existing participants or to recruit a second sample. As such, the research provides a reflective snapshot of the participants’ lives immediately before and during the pandemic, with less insight provided on how the paternal role has changed since the alleviation of the lockdown restrictions. Although the paper lacks this longitudinal reflection on fathers’ post-lockdown experiences, it is worth noting that the research offers a vital understanding of the changing relationship between paternal mental health and peer support during the pandemic.

Secondly, the participants were fathers living and working either in or locally in the city of York. As such, the sample reflects geographical particularities related to the city as uniquely affluent to the wider Yorkshire region while also being a comparatively small, Northern city compared to other cities in Yorkshire. Although the characteristics of the city were not explicitly interrogated in the study, it is worth noting that York’s cultural, socio-economic, and political climate is unique; therefore, not all findings are generalizable to participants and regions with differing circumstances. However, the participants expressed universal concerns about wanting the best for their children amidst the unprecedented challenges of the pandemic, lending this small, geographically specific study a resonance with parents across various contexts. 

Ethical approval was granted by York St John University’s Ethics Committee (RECELP00005). All participants provided their informed consent to participate in the study, and for findings to be published prior to data collection. Participants were assigned alphabetical pseudonyms to preserve anonymity. 

## 3. Findings

A key theme that ran throughout the findings was the lack of support for fathers. Within this theme, sub-themes such as: (1) the influence of gender on the relationship between fathers and informal support networks, (2) broader structural barriers to participation in informal support groups, and (3) the impact of COVID-19 pandemic on fathers’ ability to access informal support emerged. Further investigation of these themes indicated that the gendered parental division of labour influenced how fathers saw themselves and others, and their participation in informal support activities, such as playgroups. Furthermore, participants reflected on gendered, practical barriers that prevented them from accessing support. 

### 3.1. The Role of Gender

Participants suggested that gender influenced their interactions with other parents. Some of the participants experienced awkwardness or lack of a sense of belonging at play groups, which were predominantly attended by mothers, who had formed tight-knit social groups:

“*I just [felt] a bit alienated really to be honest. But I got that feeling as well when I was taking him to classes, you know, baby classes and things like that. The organisers were making a point that I was the only male in there. So, it was a bit off-putting really*”.

(O)

Fathers feel even more excluded when the mostly female groups engage in highly gendered talk. As evidenced by T, talk about childbirth and breastfeeding was fundamental to the social bonds between mothers, but the fathers could not readily participate in these conversations:

“*So, you could see that that the mothers would bond over the childbirth, birthing experience or what they did during labour. And, I obviously could not bond over that, because that’s not, I mean an experience that I went through and yeah again, there’s stuff like breastfeeding… they’d be very uncomfortable and I’d be feeling uncomfortable as well, and so I’d be maybe drawing back from that… It’s like I was going into that environment and feeling very isolated*”.

(T)

Beyond “mothering talk”, participants articulated that broader, gendered perceptions of parenting influenced their experiences. For example, local events for fathers often employed male-coded images or themes, such as football, which weren’t broadly appealing to the participants, resulting in low uptake of community-led sessions:

“*The one I went to most recently was a Father’s Day special which slightly stereotypically was football themed*”.

(G)

Participant G’s comment indicated that these broader gendered stereotypes discouraged him from attending. The leaflet strongly influenced his decision not to attend future events because it used representations of men that did not match his perception of himself as a father:

“*But there was something about the image of just a lot of men on the front and a lot of, I don’t know I’m probably just being prejudiced. But a lot of them had shaved heads and were wearing football shirts and I kind of thought I’m not sure I would fit in there [laughs]*”.

(G)

As a result, he expressed anxiety about not fitting in well with what he perceived to be the target audience. This demonstrates the importance of recognizing the range of interests and tastes that fathers have, when attempting to promote engagement.

At the informal organizational level, fathers often felt underrepresented in social media settings; mothers were more likely to frequently use communication such as WhatsApp groups. However, Participant F found that his fathers-only NCT (National Childhood Trust) WhatsApp group held frequent conversations about parenting:

“*I’ve got a WhatsApp group with all the other dads. And everyone was sort of sharing their experience of birth, at first, and sharing their first experience of being a dad at home and what everyone is doing and what problems everyone is having, how everyone is dealing with it and all that*”.

This demonstrates that fathers actively participant when groups are available. In mixed-gender WhatsApp groups, when mothers’ engagement declines, fathers are less likely to maintain the group chat and their social relationships. Participant A observes that “within two days, the mums – one of the other mums had set up a mums only group, for the NCT group, which then meant the main group just died, because dad’s are crap at talking, me included”. This echoes the broader division of social and emotional labour in heterosexual families; mothers tend to bear responsibility for social activities. 

Where other fathers in the sample returned to work, gender was highlighted as a barrier to socialising with other parents. Participant I reflects that socialising was “*a bit more awkward if you were a dad*”. Some participants suggest that their role is not taken seriously and is treated as encroaching on ‘feminine’ space and roles. Participant S describes experiencing “*weirdness and a kind of like imposition*” when participating in groups. Furthermore, mothers often use informal support networks to share frustrations about their male partners and their lack of participation in domestic labour. Therefore, complaint performs a vital discursive function for mothers in spaces such as the WhatsApp group. Participant T explains that mothers use the space to commiserate with each other, often engaging in negative talk about their partners through:

“*Mutual complaining and supporting about the support they were expecting and not receiving from the menfolk in their roles as Dads*”.

(T)

Similarly, Participant E describes feeling as though mothers that attend the same groups question his legitimacy as a father:

“*And the conversations will always end up with mums talking to other mums because in their eyes I probably don’t know anything about children or I may well be a child-minder or something*”.

Furthermore, the participants report that stereotypes regarding emotional labour persist even when they attempt to connect with predominantly male informal support networks. Although these groups were established by and for the fathers, participants commented on the superficiality of ‘male talk’, and thus, the limited efficacy of the support network. Instead of engaging in prolonged conversations about fatherhood, the groups drifted into discussion of trivial topics before dropping off altogether:

“*And there was a couple of groups but they didn’t really last very long… And it was, we just sort of fell into talking about general stuff rather than, you know, what we’d actually set the groups up for on Whatsapp*”.

(N)

They emphasise that these networks lack deeper emotional talk due to embedded perceptions and performances of masculinity. The participants frequently minimise their struggles, reflecting hegemonic masculine ideals of stoicism and ‘resilience’. For Participant M, normative beliefs about masculinity were consolidated by his military background, resulting in a tendency to ‘man up’ and to be reluctant to share his feelings and experiences:

“*I don’t think I really spoke to any person, you know, who maybe had children, or maybe they didn’t go through the similar stuff that we went through…I probably just kept everything in me, just like oh okay, maybe things will be fine, you know, just man up, and just like the army says, just man up and carry on*”.

Although these beliefs are often dismissed as stereotypical and outdated, the idea of ‘manning up’ is echoed throughout, influencing how the participants talk about their mental health. Participant D says that “*because men are different…they are less likely to ask for help, you know, men are just different.*” Many echo the idea that their emotional wellbeing is secondary to that of their partner and children and that expressing their feelings is not the ‘done thing.’ This is consolidated within the family and through interactions with the informal support network; as Participant N notes, it is “*taken for granted a little bit that dad will sort of play second fiddle to mum*”.

Additionally, Participant A notes that “*men are crap at talking to each other*”, and emotional talk is characterized as belonging to the more authentic friendships that are perceived to exist between women. Participant B acknowledges the importance of friendship, but ‘talk’ is often organically facilitated by typical masculine performances of sociability rather than being a product of more formal interactions. He expresses a cautious approach to the more formalized NCT group, highlighting how it is difficult to “*socialise with them because “[he does not want] to put too much of himself on to these folk either because they are still getting to know each other*”. Instead, he reflects on the role of traditional friendships in shared experiences of fatherhood:

“*I probably am a stereotypical bloke in that I was quite happy going to the pub or going for a bike ride with a friend and just talking with them*”.

(B)

Here, ‘deep’ talk is facilitated by traditional masculine-coded activities such as sport and drinking, rather than being the product of pressure to share in a more emotional, ‘feminine-coded’ setting. These preconceptions are consolidated by fathers who interact with predominantly female peer-support groups and find themselves feeling isolated or otherwise rejected because they struggled to participate. 

### 3.2. Practical Barriers to Participation 

Despite the cultural shift towards more egalitarian parenting practices, the participants still identified gender as creating challenges in accessing support. Many participants commented on the lack of specific information, resources, or support groups for fathers:

“*So I think the support that mothers get by default, whether they want it or not and whether they are comfortable with it or not, that’s good. And I think it’s quite efficient in terms of spotting whether mother really needs some help that she might not know about but nothing like that for fathers, unless they are really trying to seek out those opportunities*”.

(C)

As is identified by Participant C, support for mothers is rarely accompanied by similar support for fathers. Participant I states that:

“*There’s very little sort of targeted support for dads. Be it just support groups, be it dads getting together and talking about worries. Be it old dads coaching new dads, you kind of have to take it on your own initiative to do that. So, I mean there’s a couple of people at work who are having children now and I’ve sort of had to approach them because I’m friends them. I’ve had to approach them and say, ‘Hey, look I’ve gone through all of this if you need help I’m here’. But there wasn’t anything really like that for me aside from obviously, my dad*”.

Therefore, although there is a desire to engage with their peers, many fathers are prevented from doing so by a lack of opportunities, information, and resources.

Participants identified work as a key practical barrier to participation, due to organisational cultures that expect new fathers to return to work after the two-week period of paternity leave. Most playgroups run during work hours, meaning that fathers are unable to attend upon return to work. Participant D pointed out, that while mothers used the sessions as a key socializing opportunity, he did not have the opportunity because of work:

“*Baby groups, I didn’t get to go to many of them because they were all in the day, when I was back at work but my wife loved them…she was at baby groups like almost every day of the week, whether that be breastfeeding support or sensory play or baby massage, she went to a lot to try and kind of increase her social circle*”.

Therefore, fathers are disadvantaged by a lack of opportunities, because “*dads are not very good at proactively supporting each other*” (A). Other participants corroborate this, suggesting that they are often reluctant to ask each other for guidance. Participant B notes that, while his wife used WhatsApp to gain support or organize activities, he relied upon search engines rather than seek advice from peers:

“*My wife will just message all her mum friends…looking for something to do on Sunday, weather looks a bit crap… I end up Googling stuff because there isn’t that same network effect, I think you called it, which I think is right, for dads*”.

This lack of “network effect” indicates weaker social ties between fathers. It can be inferred that fathers may not construct the same types or intensity of friendships as mothers and will instead choose to seek support online. For B, this lack of support is a product of practical and socio-cultural factors; he notes that “*none of the dads that [he] knew took any extended leave*”, which limited his opportunities to socialise. 

Within friendships, it is important to acknowledge how fathers discuss their mental and emotional wellbeing. The participants suggested that they prioritise the wellbeing of their families over discussing their struggles. Participant J describes this as placing himself at the:

“*Bottom of the pecking order you know. My son is here and then my wife is here and I’ve got to look after them and then I’m last, I sort myself out last*”.

He describes the challenges that fathers must navigate in the transition to new parenthood; despite fluctuations in their wellbeing, fathers are expected to prioritise their family’s health and needs. For Participant K, this elicited challenging emotions as he felt that “*it was always harder for [his] wife. No matter what [he] was going through it wasn’t a patch on what she was going through. And in a way that delegitimised what [he] was experiencing*”. As Participant J describes, many fathers experience traumatic events during childbirth but receive no respite because “*it’s not something that’s talked about when it comes to fathers*”. The expectation is that men’s emotional wellbeing is privately managed, and that men’s mental health issues are part of the ‘backstage’ work of being a good father. Others describe the experience of new fatherhood as a total disruption; although they recognize that birth is difficult for women, participant H reflects that the ‘suddenness’ of parenthood can have significant emotional consequences for the father:

“*It’s just a shock isn’t it? You know one day you’re just living your life and then the next day all of a sudden you’ve got this baby but you’ve had no time to adjust*”.

(H)

For many fathers, this need to adjust is not recognised, and the two weeks of statutory parental leave do not provide adequate time for them to adapt. Therefore, the practical, mental, and emotional strains that impact new fathers are not addressed in informal settings.

### 3.3. The Impact of The COVID-19 Pandemic

Prior to the pandemic, most participants identified regular interaction with friends as key to their wellbeing; as A suggests, “*those relationships are really important as [the] child gets older.*” For the participants, friendships are important opportunities to seek support and to feel connected to others, but this depended on whether the friends were also parents. Some of the participants felt isolated, and found it difficult to get support from their friends because they did not share the same experiences:

“*Not many of my male friends are parents or are kind of even close to say settling down kind of cohabiting or getting married or any of the rest of it…And so, when they talk to me about it, there’s always like a mixture of awe and sort of terror [laughs] about the idea of having a life like mine*”.

(G)

Most of the participants commented that they struggled to establish meaningful, consistent support networks before the pandemic and that the lockdown regulations exacerbated this. Therefore, it is necessary to reflect on the pandemic’s impact and consider how fathers in the UK navigated the ban on in-person social gatherings. Some participants reflected on the uniqueness of the pandemic, particularly on the often isolating experience of lockdown with a young child. For participant B, competing responsibilities became difficult to manage:

“…*combination of trying to work, trying to somehow keep up your social connections with friends, and things, over Zoom, or whatever. Trying to parent. Trying to, in my case, look after my mum, who lives on her own nearby*”.

Whereas some fathers would have sought advice from their parents, the COVID-19 pandemic created a socio-cultural, generational divide within families. Some of the participants felt that their fathers would not be able to empathise. There is no scope within this paper to discuss the role that changing modes of employment and generational socio-economic divides play in shaping talk between fathers and sons. However, it is significant that Participant P identifies the shift to home working as a unique experience that he would have wished to discuss with other fathers:

“*The thing I feel I’ve not talked to people about, which I would have appreciated, being a working from home Dad during the pandemic to a newborn, that is quite a unique experience. So that was something I couldn’t speak to my Dad about [laughs] so I spoke to him about everything else but that, you know, I couldn’t, and he didn’t know how to relate to that, he’d have been back to work*”.

(P)

The challenges of the lockdown reflect existing socio-cultural tensions regarding the parental division of labour. Participant B stated that, during the pandemic, the tensions between supporting his family and adjusting to new home-working conditions meant that friendships often fell by the wayside. He acknowledges the feelings of isolation and loss that emerge from the lack of social contact, emphasising the value of these relationships for his family and his wellbeing:

“*The biggest issue I think for us, and for most people, has been the erosion of, the ability to form relationships and also to nourish those relationships in your life with family and everybody else…the hardest thing for us is that our family and friends haven’t been able to see her and see her develop in the same way we have*”.

Although interaction with family and friends might not have explicitly addressed the father’s mental health, losing this emotional outlet significantly impacts men who are experiencing mental health challenges. For Participant B, the breakdown of friendships compounds the feelings of “*failing at everything at the same time*”. As such, it is vital to acknowledge and promote bonds between fathers in the same way that friendships between mothers are valued.

## 4. Discussion

The study investigated fathers’ experiences of accessing informal support during the COVID-19 pandemic, and the impact of the COVID-19 pandemic on their mental health. Although the pandemic did present challenges to the participants’ wellbeing, these challenges were neither new nor unique to the COVID-19 pandemic. Instead, the study demonstrated that cultural perceptions of masculinity and practical barriers, such as statutory paternity leave, significantly impact fathers’ access to and engagement with informal support. Therefore, it is necessary to consider the role of hegemonic masculine constructions of fatherhood, in constructing barriers to fathers’ participation in informal support settings. Although the pandemic did present unique challenges that are explored throughout the study, the lack of support for fathers cannot solely be attributed to lockdown restrictions; rather, it is a symptom of a sociocultural landscape that fails to adequately provide for fathers as parents.

To return to Messerschmidt and Connell, ‘hegemonic masculinity’ offers a useful frame through which the participants’ experiences of fatherhood can be analysed [2]. Normative assumptions about the heterosexual parental division of labour emphasise the role of the father as being the mother’s primary supporter, rather than as a parent. Although parenting is perceived to have become more egalitarian in the UK, most participants relate their own experiences to engrained cultural assumptions about the gendered parental division of labour. This division is the product of complex assemblages of cultural beliefs and practices that reaffirm the father as breadwinner and mother as caregiver in heterosexual families. Most participants share negative experiences of participating in the often ‘female-coded’ spaces of the informal support network. Hegemonic cultural perceptions of fatherhood undermine the participants’ legitimacy, based on common assumptions that fathers are less engaged parents, and more as a babysitter. Grounded in heteronormative assumptions about the division of labour, these cultural perceptions of fatherhood prevent fathers from seeking support, information, and affirmation, and instead, create a double burden. Fathers are criticised and ostracised for being out of place and uninformed, while also being discouraged from accessing informal resources that enable them to become more knowledgeable and active parents [37].

The study found that most social activities are driven by mothers, implying that fathers who participate in these activities must navigate tacit, gendered norms around parenthood. For example, gendered talk poses a prominent barrier to many fathers when socializing online and offline; where their female counterparts discuss breastfeeding or childbirth, the participants shared feelings of discomfort and awkwardness. Mothers tend to use informal support networks to share frustrations about their male partners, which discourages fathers from participating, and further isolates them. These tensions indicate that fathers are not taken seriously or are seen as encroaching on heavily ‘feminized’ parenting spaces. Therefore, fathers are discouraged from seeking guidance and support by mothers’ complaints, because to do so would reaffirm assumptions that they are less knowledgeable or competent parents.

Fathers’ feelings of isolation are exacerbated by the lack of support that specifically targets them. Many of the participants expressed frustration at the lack of spaces that focused on fathers, particularly in comparison to mothers. This was attributed not only to the overwhelming gender divide within support groups, but also to practical barriers such as the times at which most activities are scheduled. Following the two-week statutory paternity leave period most of the participants were returned to work, meaning that they could not attend activities during the working day. This disadvantages fathers in terms of accessing informal support, whereas mothers can take up to fifty-two weeks of maternity leave and are more likely to establish support networks with other mothers. However, barriers to participation are not only practical, but are social, and are rooted in heavily gendered dynamics between mothers and fathers. Where support groups were advertised as mixed gender, the fathers expressed how unwelcome they felt, and how the focus often returned to mothers, due to the predominantly female demographic of the groups. This demonstrates that, within these spaces, fathers are often characterised as ancillary or secondary to mothers. 

When father specific spaces are provided, the study found that the wording used to advertise the events does not promote participation, because it does not indicate that the activity would provide a supportive space for deep fatherhood discussion. Participants also found the use of stereotypically ‘male’ themes to dissuade them from attending activities because it did not feel inclusive. This indicates the importance of recognizing fathers’ diverse interests and needs when advertising activities. Using male-coded language and images relies on familiar masculine tropes that are not representative of all the men that they are attempting to engage. Arguably, this is a significant issue that needs recognition; if fathers do not feel represented, they may be discouraged from participating and building productive relationships with other parents.

The study demonstrates that mothers do not always get the support that they need, but that they are more proactive in seeking and establishing support networks, such as the NCT WhatsApp. Fathers on the contrary seem to be less proactive in asking for support or setting up such support networks, often choosing to use search engines to seek guidance, or otherwise internalising their wellbeing concerns. Therefore, it is a broader societal need to understand and support fathers in establishing strong, long-lasting friendship groups. However, the participants in the sample indicated that they struggle to engage in supportive talk about parenting, even when there are opportunities to do so. They found that group conversations often drift into other, irrelevant topics. These conversations reaffirm popular cultural tropes regarding men’s inability to engage in meaningful conversations and demonstrate the gendering not only of the more material aspects of the parental division of labour, but also of the emotional labour of parenting talk. The participants expressed that they did value supportive relationships with other fathers but emphasised that these networks lack ‘feminine’ depth and empathy, reinforcing familiar tropes of masculinity [2]. 

Participants struggled to treat themselves with empathy, instead enacting hegemonic, masculine ideals of stoicism by downplaying their struggles in relation to their partners’ experiences. This was common when participants talked about their wellbeing. Often, they foreground their partner or children and talk generally or flippantly about their mental health, reducing its importance and preserving the stoic façade they are required to perform to their peers. Socio-cultural expectations encouraged the participants to consider their emotional wellbeing to be secondary to that of their family, because they are not perceived to experience mental health challenges to the same extent. Furthermore, participants find it difficult to navigate fatherhood and their mental health due to the social construction of taboos around paternal wellbeing [38] and the perceived ‘ease’ with which fathers are seen to transition into fatherhood.

It is worth noting that increasing the provision of informal support networks may not immediately correlate to greater uptake or more in-depth engagement. For prospective members, it can take time to establish friendships and feel comfortable sharing deeper wellbeing experiences, particularly where cultural perceptions of vulnerability and masculinity persist. The findings suggest that fathers are more likely to engage in deeper discussions about fatherhood and mental health if this is not the main reason for meeting up with other people, and another activity acts as a precursor. Corresponding with Darwin et al. [10], the participants are more comfortable talking during activities that organically encourage conversation rather than in settings where emotional talk is the goal. These preconceptions are consolidated by fathers who interact with predominantly female peer- groups and feel isolated or otherwise rejected because they struggle to participate. 

Prior to the COVID-19 pandemic, participants struggled to establish meaningful relationships, but the situation was made worse by the ban on in-person social gatherings. Where fathers identified otherwise productive opportunities to engage with other parents, the pandemic created material and legal barriers to in-person interaction. Where fathers rely on their parents for advice, the uniqueness of the pandemic meant that their parents may not be able to understand their situation and may not be able to offer relevant support. The pandemic provided an extra challenge to those that became fathers during the lockdown and had to balance looking after the child and working from home. As a result, many fathers in the sample dismissed their own struggles and prioritised the wellbeing of the family. 

The study suggests that, even though fathers recognise the value of engaging with informal support networks, they are not always readily available, due to an emphasis on mothers, and the socially constructed differentiations between the roles of mother and father. Where support is available, mothers tend to be more proactive in accessing it than fathers. On the other hand, fathers are often discouraged from proactively seeking support due to gendered stereotypes about parenting and practical barriers such as work. Although the pandemic made these issues apparent, there is an immediate need to tackle the pervasive and deeply engrained stereotypes around fatherhood that existed prior to the emergence of COVID-19 pandemic.

## 5. Conclusions

The study demonstrated that informal support networks are important to fathers as a means of sharing advice and experiences. However, the focus on mothers and lack of specific support for fathers remains a key challenge. To support fathers, father-specific support, information, and resources must be increased, and recognize the diverse needs of fathers. These and existing support groups should be accessible to fathers, by running outside of the working day. Predominantly, there is a need for the government to review the current paternity leave policies to ensure that fathers have a longer independent period of leave that will enable them to be more active participants in the lives of their children. By providing this support, policy will encourage a wider cultural acknowledgement of fathers as active parents. In the long term, this will help to dismantle gendered stereotypes about parenting that currently act as barriers and prevent fathers from seeking the support that they need. 

Further research must seek a more nuanced understanding of the issues that impact the social lives and wellbeing of fathers. Similarly, there is a need to investigate the impact that changing working patterns, economic conditions, and generational divides have on fatherhood and fathers’ mental health. Finally, further research should consider the role of informal support mechanisms in upholding the gendered parental division of labour.
